# Overexpression of FOXG1 contributes to TGF-*β* resistance through inhibition of p21^WAF1/CIP1^ expression in ovarian cancer

**DOI:** 10.1038/sj.bjc.6605316

**Published:** 2009-09-15

**Authors:** D W Chan, V W S Liu, R M Y To, P M Chiu, W Y W Lee, K M Yao, A N Y Cheung, H Y S Ngan

**Affiliations:** 1 Department of Obstetrics and Gynaecology, LKS Faculty of Medicine, University of Hong Kong, Hong Kong SAR, P. R. China; 2 Department of Biochemistry, LKS Faculty of Medicine, University of Hong Kong, Hong Kong SAR, P. R. China; 3 Department of Pathology, LKS Faculty of Medicine, University of Hong Kong, Hong Kong SAR, P. R. China

**Keywords:** FOXG1, p21WAF1/CIP1, TGF-*β*, ovarian cancer

## Abstract

**Background:**

Loss of growth inhibitory response to transforming growth factor-*β* (TGF-*β*) is a common feature of epithelial cancers. Recent studies have reported that genetic lesions and overexpression of oncoproteins in TGF-*β*/Smads signalling cascade contribute to the TGF-*β* resistance. Here, we showed that the overexpressed FOXG1 was involved in attenuating the anti-proliferative control of TGF-*β*/Smads signalling in ovarian cancer.

**Methods:**

FOXG1 and p21^WAF1/CIP1^ expressions were evaluated by real-time quantitative reverse-transcription polymerase chain reaction (RT–PCR), western blot and immunohistochemical analyses. The effect of FOXG1 on *p21*^*WAF1/CIP1*^ transcriptional activity was examined by luciferase reporter assays. Cell lines stably expressing or short hairpin RNA interference-mediated knockdown FOXG1 were established for studying the gain-or-loss functional effects of FOXG1. XTT cell proliferation assay was used to measure cell growth of ovarian cancer cells.

**Results:**

Quantitative RT–PCR and western blot analyses showed that *FOXG1* was upregulated and inversely associated with the expression levels of *p21*^*WAF1/CIP1*^ in ovarian cancer. The overexpression of *FOXG1* was significantly correlated with high-grade ovarian cancer (*P*=0.025). Immunohistochemical analysis on ovarian cancer tissue array was further evidenced that FOXG1 was highly expressed and significantly correlated with high-grade ovarian cancer (*P*=0.048). Functionally, enforced expression of FOXG1 selectively blocked the TGF-*β*-induced *p21*^*WAF1/CIP1*^ expressions and increased cell proliferation in ovarian cancer cells. Conversely, FOXG1 knockdown resulted in a 20–26% decrease in cell proliferation together with 16–33% increase in *p21*^*WAF1/CIP1*^ expression. Notably, FOXG1 was able to inhibit the *p21*^*WAF1/CIP1*^ promoter activity in a p53-independent manner by transient reporter assays.

**Conclusion:**

Our results suggest that FOXG1 acts as an oncoprotein inhibiting TGF-*β*-mediated anti-proliferative responses in ovarian cancer cells through suppressing *p21*^*WAF1/CIP1*^ transcription.

The transforming growth factor-*β* (TGF-*β*) family consists of multifunctional cytokines that control a wide variety of biological activities including cell proliferation, differentiation, apoptosis, cell adhesion, extracellular matrix formation, immune cell regulation and embryonic development through activating TGF-*β* receptors and Smad transducer proteins (Hu *et al*, 1998; Massague, 1998). The complicated role of TGF-*β* in mediating these cellular activities depends on cell types, growth environment, concentration of ligands and the presence of other growth factors (Hu *et al*, 1998; Massague, 1998; Savage-Dunn, 2005). Inhibition of cell proliferation is one of the biological effects of TGF-*β* on normal epithelial cells, suggesting that TGF-*β* acts as a tumour suppressor (Moses *et al*, 1987). Loss of autocrine activity and/or responsiveness to TGF-*β* is frequently found in human cancers during tumour progression. Mutations, deletions or methylation of members of TGF-*β* signalling pathway leading to TGF-*β* resistance in human cancers have been extensively reported. For examples, decreased expressions or mutations in *TGF-β R1*, *TGF-β RII* and *Smad4* have been frequently detected in a number of human cancers. (Eisma *et al*, 1996; Kim *et al*, 1996; Ko *et al*, 1998; Korchynskyi *et al*, 1999; Matsushita *et al*, 1999; Venkatasubbarao *et al*, 2000; Maurice *et al*, 2001; Paik *et al*, 2003; Sakaguchi *et al*, 2005; Perttu *et al*, 2006; Zhong *et al*, 2006).

On the other hand, elevated expression of proto-oncogenes or oncogenes may also cause TGF-*β* resistance in tumour cells. For example, upregulation of the c-Ski and SnoN represses the growth inhibitory function of the Smad proteins (He *et al*, 2003); increased expression of the oncoprotein HER2/Neu activates *Smad7* transcription (Dowdy *et al*, 2003), and overexpression of BCL6 disrupts the Smad-p300 interaction and represses the transcriptional activity of Smad4 (Wang *et al*, 2008). Moreover, increased expression of the *EWS/Fli1* oncogene, the Tax oncoprotein from HTLV-1, the E1A oncoprotein of DNA virus and the E7 oncoprotein of HPV resulted in reduced TGF-*β* responsiveness (Datta and Bagchi, 1994; Hahm *et al*, 1999; Mori *et al*, 2001; Lee *et al*, 2002). Given the important tumour suppressor functions of the TGF-*β* pathway, it is expected that other oncoproteins could promote tumourigenesis by counteracting this pathway.

FOXG1, also known as brain factor-1, is a member of Forkhead box family of transcription factors (Arden, 2004; Obendorf *et al*, 2007). FOXG1 contains a highly conserved DNA-binding domain, which binds to specific DNA sequences and regulates gene expression (Yao *et al*, 2001). Recent studies have shown that FOXG1 acts as a negative regulator of TGF-*β* signalling pathway by specifically binding to the Smad MH2 domain and associates with Smad -1, -2, -3 and -4 (Dou *et al*, 2000; Seoane *et al*, 2004). This association blocks the binding of Smad proteins to DNA and results in the inhibition of TGF-*β* signalling (Rodriguez *et al*, 2001). In addition, FOXG1 has been shown to inhibit expression of the cyclin-dependent kinase (CDK) inhibitor *p21*^*WAF1/CIP1*^, which is normally transcriptionally activated by TGF-*β* signalling, in glioblastoma and the neuroepithelium (Pardali *et al*, 2000; Seoane *et al*, 2004). However, the functions of FOXG1 in other human cancers remain unclear.

In this study, we reported that the FOXG1 was overexpressed in ovarian cancer. The overexpressed FOXG1 was significantly correlated with high-grade ovarian cancer. More importantly, we showed that the increased expression of FOXG1 significantly suppressed the expression of p21^WAF1/CIP1^ and increased cell proliferation of ovarian cancer cells. Altogether, these findings suggest that FOXG1 was associated with attenuating the TGF-*β* anti-proliferative response in ovarian cancer.

## Materials and methods

### Clinical samples and cell lines

Sixty-seven ovarian cancer tissues and 49 normal ovarian tissues were obtained from the Department of Obstetric and Gynaecology at Queen Mary hospital. The histological subtypes and disease stages of the ovarian tumours were classified according to the International Federation of Gynaecology and Obstetrics (FIGO) criteria. All the clinical specimens used in this study were approved by the local institutional ethics committee (Institutional Review Board number: UW05-143 T1806). Four immortalised human ovarian surface epithelial (HOSE) cells were used in this study: HOSE 6-3, HOSE 10-2, HOSE 11-12, HOSE 17-1 and HOSE 11-24 (from Prof George Tsao, the University of Hong Kong). Ovarian cancer cell lines OV2008, C13^*^, A2780s, A2780cp (gift from Prof Benjamin Tsang, University of Ottawa), OVCAR3, SKOV3, OV420, OV429 and OV433 (American Type Culture Collection, Rockville, MD, USA) were used in this study. All were grown at 37°C in 5% CO_2_ in minimum essential medium or Dulbecco's modified Eagle medium supplemented with 10% foetal bovine serum.

### Plasmids and cell transfection

The pCMV2-Flag-FOXG1-expressing plasmid (gift from Dr Stefano Stifani from McGill University, Montreal, Quebec, Canada) was used for ectopic expression of Flag-tagged FOXG1. The short hairpin RNA interference (shRNAi) targeting FOXG1 (target sequence: TCTGTCCCTCAACAAGTGC) was ligated into pTER vector (gift from Dr Marc van de Wetering, Centre for Biochemical Genetics, the Netherlands) to generate pTER-shFOXG1 plasmid. A human mutant *p21*^*WAF1/CIP1*^ promoter luciferase construct (pWWP) containing a truncated *p21*^*WAF1/CIP1*^ promoter with deleted p53-binding sites (gift from Dr Mark Feitelson, Mercer Laboratory, Thomas Jefferson University, Philadelphia, PA, USA) was used for luciferase reporter assay. LipofectAMINE 2000 (Invitrogen Life Technologies, Carlsbad, CA, USA) and Fugene6 Transfection Reagent (Roche Biosciences, Indianapolis, IN, USA) were used for cell transfection according to the manufacturer's instructions. The pcDNA3 and pTER empty vectors were used as mock transfection, respectively, in enforced expression and knockdown assays, whereas pRL-SV40 (Promega, Madison, WI, USA) was served as an internal control in luciferase reporter assay. Stably overexpressed Flag-tagged FOXG1 or FOXG1 knockdown clones were established by drug selection using G418 at 400 *μ*g ml^−1^ for 2 weeks or puromycin at 2 *μ*g ml^−1^ for 1 week, respectively. Positive clones were randomly chosen for cell number expansion and verified by western blot analysis.

### Quantitative and semi-quantitative reverse-transcription polymerase chain reaction

Total RNA from each cell line was prepared by TRIzol reagent (Invitrogen). First strand cDNA was synthesised by random hexamers and Taqman reverse transcription reagent kit (Applied Biosystems, Foster City, CA, USA). For real-time quantitative reverse-transcription polymerase chain reaction (RT–PCR) (Q-PCR), the amount of *FOXG1* and *p21*^*WAF1/CIP1*^ genes were quantified by TaqMan Gene Expression Assays and in an ABI 7700 system (Applied Biosystems) using the *FOXG1* and *p21*^*WAF1/CIP1*^ primers and probe from Applied Biosystems (*FOXG1*, assay ID: Hs00702391_s1; *p21*^*WAF1/CIP1*^, assay ID: Hs00355782; *GAPDH*, assay ID: Hs99999905_m1). Each sample was performed in triplicate and normalised with human GAPDH (assay ID: Hs99999905_m1; Applied Biosystem). For semi-quantitative RT–PCR, the *p21*^*WAF1/CIP1*^ mRNA level was evaluated by a pair of primers (*p21*^*WAF1/CIP1*^*-Sense* 5′-ACCATGTGGACCTGTCACTGTCTT-3′ and *p21*^*WAF1/CIP1*^*-Antisense* 5′-AGAAGATGTAGAGCGGGCCTTTGA-3′) with the following conditions for 30–35 cycles: denaturation at 94°C for 30 sec, annealing at 58°C for 30 sec and extension at 72°C for 30 sec. The relative amount of *p21*^*WAF1/CIP1*^ was normalised using *GAPDH* mRNA with the following primers (*GAPDH-Sense*: 5′-ACGCATTTGGTCGTATTGGG-3′ and *GAPDH-Antisense*: 5′-TGATTTTGGAGGGATCTCGC-3′) and amplification at 25 cycles: denaturation at 94°C for 30 sec, annealing at 55°C for 30 sec and extension at 72°C for 1 min.

### Immunohistochemical and western blot analyses

Immunohistochemical staining for FOXG1 was performed on an ovarian cancer tissue array (OVC961) (Pantomics Inc, San Francisco, CA, USA). The section was immunostained with primary rabbit polyclonal anti-FOXG1 antibody (Abcam Inc, Cambridge, MA, USA) in 1 : 20 dilution. For negative controls, the primary antibody was replaced with Tris-buffered saline. The intensity of staining was scored as 0 (negative), 1+ (faint), 2+ (moderate), 3+ (strong) and 4+ (marked).

For western blot analysis, samples containing equal amounts of protein were separated by SDS–PAGE and electroblotted onto Hybond-P membranes (Amersham Pharmacia Biotech, Cleveland, OH, USA). Blots were blotted with 5% skimmed milk and analysed by immunoblotting with antibodies specific for anti-FOXG1 (Abcam), anti-Flag, anti-*β*-actin and anti-phosphoserine (Sigma Chemical Co, St Louis, MO, USA), anti-p21^WAF1/CIP1^, anti-Smad3 and anti-phospho-Smad3 (Cell Signalling Technology, Darvers, MA, USA) and anti-Histone H1 (Santa Cruz Biotechnology Inc, Santa Cruz, CA, USA). Blots were then incubated with goat anti-mouse or anti-rabbit secondary antibodies conjugated to horseradish peroxidase (Amersham) and visualised by enhanced chemiluminescence (ECL) (Amersham).

### Subcellular fractionation and phosphorylation studies

The cellular localisation of endogenous FOXG1 or Flag-tagged FOXG1 in ovarian cancer cells was examined by western blot analysis on subcellular extracts prepared from NE-PER nuclear and cytoplasmic extraction reagents according to the manufacturer's protocol (Pierce Biotechnology, Rockford, IL, USA). The serine phosphorylation status of FOXG1 in ovarian cancer cells was analysed according to Regad *et al* (2007). Briefly, ovarian cancer cells were collected in NET lysis buffer (50 mM Tris, pH 7.4, 150 mM NaCl and 5 mM EDTA) with 1% NP40, pH 8.0, 0.1 mM PMSF and 1 mM Complete TM protease inhibitor cocktail (Roche). FOXG1 or Flag-tagged FOXG1 was immunopreciptated from 500 ng whole cell lysates by 0.5 *μ*g of anti-FOXG1 (Santa Cruz) by incubation with Protein A/G Plus-Agarose beads (Santa Cruz) at 4^°^C. Immunoprecipitates on the beads were washed four times with NET lysis buffer, followed by elution using SDS sample buffer (Cell Signalling) and denature by boiling before electrophoresis.

### Luciferase reporter assay

Cells were seeded in 24-well plates and transiently transfected with various amounts of pCMV2-Flag-FOXG1 vector with pWWP-luciferase reporter construct. Cells were incubated with the transfection reagents for at least 24 h and were then replaced with fresh medium containing 100 pM TGF-*β*. After 5 h of TGF-*β* incubation, cells were lysed for luciferase activity analysis using the Dual-Luciferase Reporter Assay System (Promega). The transfection efficiency was normalised with *Renilla* luciferase activity. All experiments were repeated three times.

### Cell viability analysis

Cell viability was measured by Cell Proliferation kit II (XTT) for 5 days according to the manufacturer's instructions (Roche). The experiment was performed in triplicate for each time point. Human TGF-*β*1 was purchased as lyophilised samples from R&D systems (Minneapolis, MN, USA). Sterile 4 mM HCl (Merck, Darmstadt, Germany) containing 0.1% (v/v) bovine serum albumin (Pierce) was added to prepare a stock solution of 10 *μ*g ml^−1^.

### Statistical analysis

Student's *t* test (for parametric data) and the Mann–Whitney test (for non-parametric data) were used. Correlation of gene expression was analysed using Spearman's rho non-parametric statistics. Statistical analyses on clinicopathological correlation were performed using the SPSS version 13.0 software (SPSS, Chicago, IL, USA). A *P*-value was considered significant when <0.05.

## Results

### Overexpression of FOXG1 in ovarian cancer

**Figure 1:**
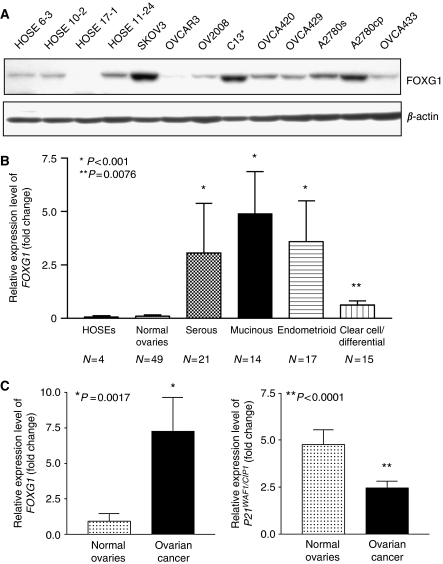
*FOXG1* is underexpressed and is inversely correlated with *p21*^*WAF1/CIP1*^ in normal ovaries and ovarian cancer tissues. (**A**) Western blot analysis showed the expression of FOXG1 in ovarian cancer cell lines and immortalised normal ovarian epithelial cell lines (HOSEs). (**B**) The relative expression levels of *FOXG1* was evaluated by quantitative RT–PCR on HOSEs, normal ovaries and four histological subtypes of ovarian cancers (^*^P<0.001; ^**^P=0.0076). *N* is the number of subtype cases. (**C**) Quantitative RT–PCR showed the expression level of *FOXG1* and *p21*^*WAF1/CIP1*^ in normal ovaries and ovarian cancer tissues. The values were obtained by mean±s.e.m. (^*^P=0.001; ^**^P<0.0001).

Our preliminary data using cDNA microarray analysis has shown that the expression of *FOXG1* in ovarian cancer cell lines was 2.2-folds higher than that in HOSE cells (Liu *et al*, unpublished data). In this study, we found that four out of nine ovarian cancer cell lines (SKOV3, C13^*^, A2780s and A2780cp) expressed relatively higher levels of FOXG1 as compared with four HOSEs cell lines by western blotting (Figure 1A). To further confirm the upregulation of *FOXG1* in ovarian cancer, we evaluated the expression status of *FOXG1* in ovarian cancer tissues (*n*=67) and normal ovarian tissues (*n*=49) by Q-PCR analysis. On the basis of comparative CT method using *GAPDH* as the endogenous control, very low expression level of *FOXG1* was detected in normal ovaries and HOSEs (Figure 1B). Conversely, *FOXG1* expression was significantly higher in serous, mucinous, endometrioid (*P*<0.001) and clear cell/undifferentiated (*P*=0.0076) subtypes of ovarian tumours when compared with the normal ovaries (Figure 1B).

As earlier studies have shown that FOXG1 is able to block the transcription of p21^WAF1/CIP1^ by counteracting TGF-*β*-induced signalling pathway and hence promoting cell proliferation (Seoane *et al*, 2004; Adesina *et al*, 2007a), it would be interesting to examine the gene expression of *p21*^*WAF1/CIP1*^ in normal ovaries and ovarian cancer tissues. Using Q-PCR analysis, we found that *p21*^*WAF1/CIP1*^ showed higher expression levels in normal ovarian tissues when compared with ovarian tumour (*P*<0.0001) (Figure 1C). In addition, the expression status of *p21*^*WAF1/CIP1*^ was inversely associated with *FOXG1* (Figure 1C). However, no statistical significant negative correlation was found for *FOXG1* and *p21*^*WAF1/CIP1*^ expressions using non-parametric Spearman rho test (*r*=−0.05, *P*>0.05).

### Clinicopathological correlation of FOXG1 and ovarian cancer

On clinicopathological correlation, we found that the *FOXG1* overexpression (>1.5-fold) in ovarian cancer was significantly correlated with high-grade tumour (*P*=0.025) (Table 1). However, there was no association with any histological subtypes of ovarian cancers (serous, mucinous, endometrioid and clear cell), tumour stage, recurrence and age (Table 1), as well as the patient's survival (data not shown). This indicates that the overexpression of *FOXG1* does not link to any specific subtypes, tumour stage and patient's survival but is involved in high-grade tumours of ovarian cancer.

### Immunohistochemical analysis of FOXG1 expression in ovarian cancer tissue array

**Figure 2:**
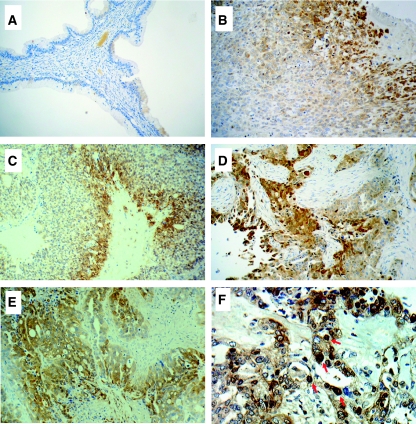
Immunohistochemical analyses of FOXG1 expression in ovarian cancer tissues. FOXG1 staining in (**A**) benign, (**B**) clear cell/undifferentiated, (**C**) mucinous, (**D**) serous and (**E**) endometrioid ovarian cancers. (**F**) Cytoplasmic and nuclear localisation of FOXG1. Arrow, nuclear localisation of FOXG1. Magnification: × 200 (**A**, **B**, **C**, **D** and **E**), and × 400 (**F**).

Expression of FOXG1 was also assessed by immunohistochemical staining in ovarian cancer tissue array (OVC961) (Pantomics Inc), which has 31 cases of epithelial ovarian cancer with different histological subtypes (serous, mucinous, endometrioid, clear cell/undifferentiated) and 6 cases of normal and benign tumour tissues. FOXG1 protein expression was evaluated as 0 (negative), 1+ (faint), 2+ (moderate), 3+ (strong) and 4+ (marked). FOXG1 staining was observed in 80.6% (25 out of 31) of ovarian carcinomas examined whereas positive staining was rarely observed in the epithelial cells of normal ovarian and benign tumour tissues (Figure 2A) (Table 2). Positive staining of FOXG1 was observed in 100.0% (9 out of 9) serous, 66.7% (4 out of 6) mucinous, 84.6% (11 out of 13) endometrioid and 33.3% (1 out of 3) clear cell/undifferentiated ovarian cancer (Table 2) (Figure 2B–E). Of 29 ovarian cancer cases with tumour grade and stage scores, high intensity (>3+) of FOXG1 was significantly correlated with high-grade tumour (*P*=0.048) but no association was found for tumour stage (data not shown). This finding was consistent with the quantitative RT–PCR data. Besides, FOXG1 expression was mainly localised in the cytoplasm, but nuclear localisation of FOXG1 was also detected (Figure 2F), suggesting that FOXG1 exhibits nucleocytoplasmic shuttling and has different functions in cells.

### The effect of TGF-*β* on ovarian cancer cells

To examine whether the overexpressed FOXG1 exerts inhibitory role on TGF-*β*-induced p21^WAF1/CIP1^ expression similar to earlier reports (Seoane *et al*, 2004; Adesina *et al*, 2007a), several ovarian cancer cell models were used. Genetic and/or epigenetic lesions usually deregulate TGF-*β*-mediated cell growth inhibitory effects in various human cancers, including ovarian cancer (Sakaguchi *et al*, 2005). To exclude ovarian cancer cell lines with malfunctioned TGF-*β* signalling, we tested the TGF-*β*/Smad signalling component, Smad3 and the p21^WAF1/CIP1^ gene induction under TGF-*β* treatment for the selected cell lines. To test the effect of TGF-*β* on ovarian cancer cell lines, cells were cultured in the presence or absence of 100 pM TGF-*β* in serum-free medium for 5 h and were then collected for immunoblotting. As Smad3 is one of the key effectors of TGF-*β* signalling, the levels of Smad3 and phosphorylated Smad3, which represent the activated form of Smad3, were examined.

**Figure 3:**
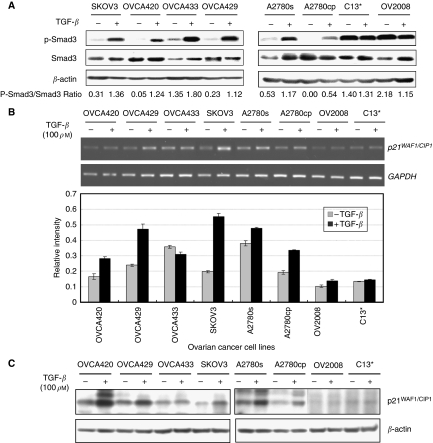
Effects of TGF-*β* on ovarian cancer cell lines. (**A**) Activation of Smad3 on TGF-*β* treatment. Ovarian cancer cell lines were cultured in the presence (+) or absence (−) of 100 pM TGF-*β* in serum-free medium for 5 h and were then collected for immunoblotting using antibodies specific to Smad3 and phosphorylated form of Smad3. Loading was compared with the internal control using antibody against *β*-actin. Semi-quantitative RT–PCR (**B**) and western blot (**C**) analyses showed the induction of p21^WAF1/CIP1^ expression by TGF-*β*. For semi-quiantitative RT–PCR analysis, the relative *p21*^*WAF1/CIP1*^ mRNA level normalised by the level of *GAPDH* was then plotted and presented as a bar chart.

On TGF-*β* treatment, activation of Smad3 was observed in OVCA420, OVCA429, OVCA433, SKOV3, A2780s and A2780cp cells, but activation of Smad3 was not observed in OV2008 and C13^*^ after TGF-*β* treatment (Figure 3A). Moreover, by semi-quantitative RT–PCR and western blot analyses, induction of p21^WAF1/CIP1^ expression was observed in OVCA420, OVCA429, SKOV3, A2780s and A2780cp (Figure 3B and C). As these five cell lines also showed Smad3 activation on TGF-*β* treatment, we believe that these cell lines are still responsive to the inhibitory effect of TGF-*β* signalling. However, OVCA433, which showed Smad3 activation in the presence of TGF-*β*, failed to show p21^WAF1/CIP1^ induction after TGF-*β* addition. It is suspected that the TGF-*β* signalling pathway downstream of Smad3 has been disrupted in OVCA433. Taken together, five of the ovarian cancer cell lines studied (OVCA420, OVCA429, SKOV3, A2780s and A2780cp) acquire a functional TGF-*β* signalling pathway and are able to drive the transcriptional activation of p21^WAF1/CIP*1*^ on TGF-*β* treatment.

### FOXG1 inhibits *p21*^*WAF1/CIP1*^ promoter activity on TGF-*β* treatment

**Figure 4:**
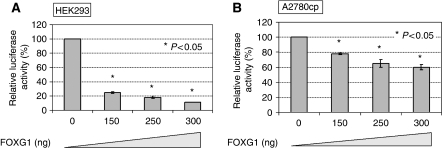
FOXG1 inhibits p21^WAF1/CIP1^ promoter activity on TGF-*β* treatment. (**A**) HEK293T, (**B**) A2780cp cells were transfected with fixed amount of the reporter plasmids pWWP and pRL-SV40 and varied amounts of pCMV2-Flag-FOXG1 and empty vector pCMV2. 100 pM TGF-*β* was added 24 h after transfection and was incubated for another 5 h before measuring the luciferase activity. The promoter activity of empty vector pCMV2 transfection was set to be 100% and the promoter activity with FOXG1 plasmids addition was expressed as a percentage relative to empty vector control.

To determine whether FOXG1 regulates *p21*^*WAF1/CIP1*^ expression at the transcriptional level, luciferase reporter assay was conducted. As it has been well known that *p21*^*WAF1/CIP1*^ is a downstream target of p53, truncated *p21*^*WAF1/CIP1*^ promoter without p53-binding site (pWWP) was used to ensure that the change of *p21*^*WAF1/CIP1*^ promoter activity was independent of p53 regulation. On transfection of *FOXG1* at increasing amounts (0, 150, 250 and 300 ng) and treatment of TGF-*β* (100 pM), the relative luciferase activity of *p21*^*WAF1/CIP*^ was reduced from 100% to 25%, 19% and 11%, respectively, in HEK293T cells (*P*<0.05), and from 100% to 79%, 65% and 60%, respectively, in A2780cp cells (*P*<0.05) (Figure 4A and B). These data suggest that FOXG1 regulates the expression of *p21*^*WAF1/CIP1*^ at the transcriptional level in HEK293T as well as A2780cp ovarian cancer cells.

### Enforced expression of FOXG1 inhibits p21^WAF1/CIP1^ induction and increases cell proliferation on TGF-*β* treatment

**Figure 5:**
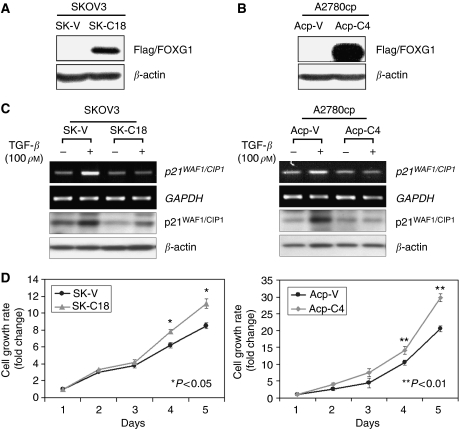
Enforced expression of FOXG1 blocks the induction of p21^WAF1/CIP1^ and promotes cell proliferation on TGF-*β* treatment. (**A, B**) Western blot analysis showed the expression of Flag-tagged FOXG1 in two stable clones, SK-C18 and Acp-C4, of SKOV3 and A2780cp ovarian cancer cell lines, respectively. SK-V and Acp-V are their corresponding vector controls. (**C**) The vector controls and Flag-tagged FOXG1 stable clones of SKOV3 (left) and A2780cp (right) were cultured in the presence (+) or absence (−) of 100 pM TGF-*β* for 24 h. The p21^WAF1/CIP1^ mRNA and protein levels were analysed by semi-quantitative RT–PCR and western blot analyses, respectively. (**D**) The vector controls and Flag-tagged FOXG1 stable clones of SKOV3 (left) and A2780cp (right) were cultured in media containing 10 pM TGF-*β* for 5 days. XTT assay showed lower cell proliferation rate in FOXG1-overexpressed clones (38% for SK-C18, *P*<0.05; and 48% for Acp-C4, *P*<0.01) as compared with their vector controls.

To investigate the inhibitory function of FOXG1 on TGF-*β*-induced p21^WAF1/CIP1^ expression and cell proliferation, we first generated Flag-tagged FOXG1 stable-expressing clones from two ovarian cancer cell lines, SKOV3 and A2780cp (Figure 5A and B). On TGF-*β* treatment, a 2.6-fold and 1.5-fold induction of p21^WAF1/CIP1^ mRNA and protein levels were observed in SKOV3 and A2780cp vector controls, respectively (Figure 5C). However, there was no change in p21^WAF1/CIP1^ levels on TGF-*β* treatment for two stable clones expressing Flag-tagged FOXG1 of both cell lines (SK-C18 and Acp-C4) (Figure 5C). This suggests that the overexpressed FOXG1 suppresses TGF-*β*-induced p21^WAF1/CIP1^ expression. We next examined the effect of enforced FOXG1 expression on cell growth using *in vitro* proliferation assay (XTT assay). In the presence of 10 pg TGF-*β*1 in cell culture medium, enforced expression of FOXG1 in both stable clones exhibited a higher cell proliferation rate (38% for SK-C18, *P*<0.05; and 48% for Acp-C4, *P*<0.01) as compared with their vector control cell lines (Figure 5D). These results suggest that FOXG1 promotes the cell proliferation of ovarian cancer cells, which is consistent with the role of FOXG1 in the inhibition of anti-proliferative effect of TGF-*β* reported in earlier studies.

### Depletion of FOXG1 sensitises TGF-*β*-mediated p21^WAF1/CIP1^ induction and cell growth inhibition

**Figure 6:**
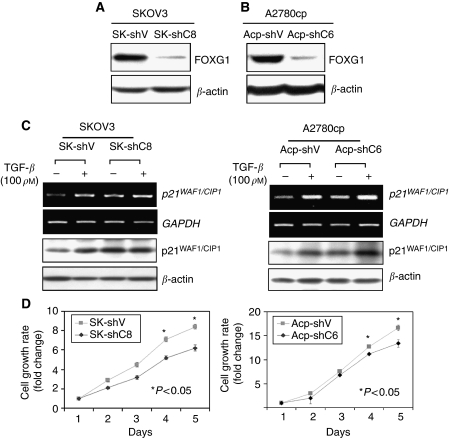
Depletion of FOXG1 sensitises TGF-*β* mediated of p21^WAF1/CIP1^ induction and cell growth inhibition. (**A, B**) Western blot analysis showed the reduction of endogenous FOXG1 by FOXG1 shRNAi plasmid, pTER-shFOXG1, SKOV3 and A2780cp ovarian cancer cell lines, respectively. SK-shV and Acp-shV are their corresponding vector controls. (**C**) The vector controls and FOXG1 stable knockdown clones of SKOV3 (SK-shV and SK-shC8) (left), and A2780cp (Acp-shV and Acp-shC6) (right) were cultured in the presence (+) or absence (−) of 100 pM TGF-*β* for 24 h. The p21^WAF1/CIP1^ mRNA and protein levels were analysed by semi-quantitative RT–PCR and western blot analyses, respectively. (**D**) FOXG1 knockdown clones of SKOV3 (SK-shV and SK-shC8) (left), and A2780cp (Acp-shV and Acp-shC6), cultured in the medium supplemented with 10 pM TGF-*β* for 5 days. XTT assay shown higher cell proliferation rate in FOXG1-depleted clones (26% for SK-shC8 and 20% for Acp-shC6, *P*<0.05) as compared with their vector controls.

To further confirm the inhibitory effect of FOXG1 on TGF-*β* signalling in ovarian cancer cells, we used the vector-based RNAi technique and successfully knockdown >75% endogenous FOXG1 in an FOXG1 overexpressing cell lines, SKOV3 and A2780cp (Figure 6A and B). On TGF-*β* treatment, additional increase of p21^WAF1/CIP1^ expressions was observed in the FOXG1-depleted clones of SKOV3 (16% increase in SK-shC8) and A2780cp (33% increase in Acp-shC6) cells as compared with empty vector controls (Figure 6B). XTT assay also demonstrated that depletion of FOXG1 could reduce 26% (SK-shC8) and 20% (Acp-shC6) cell proliferation rate (*P*<0.05) in the presence of 10 pg TGF-*β* in SKOV3 and A2780cp cells, respectively (Figure 6C). These data further support that FOXG1 counteracts TGF-*β*-mediated cell growth arrest through regulation of p21^WAF1/CIP1^ induction.

### Inhibition of p21^WAF1/CIP1^ induction because of increased nuclear localisation of FOXG1

**Figure 7:**
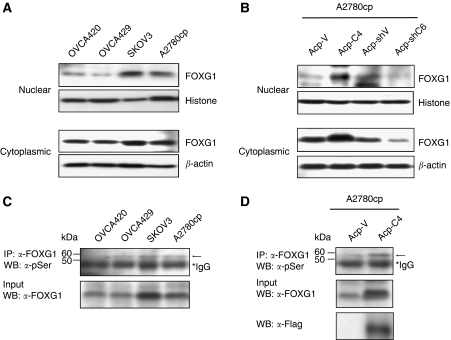
Subcellular localisation and phosphorylation of FOXG1. Western blot analysis showed the expression levels of cytoplasmic and nuclear FOXG1 in ovarian cancer cell lines (OVCA420, OVCA429, SKOV3 and A2780cp) (**A**), and the expression of Flag-tagged FOXG1 and FOXG1 in Flag-tagged FOXG1 stable clone (Acp-C4) and FOXG1 knockdown clone (Acp-shC6) of A2780cp (**B**). Histone H1 and *β*-actin were used as internal controls of nuclear and cytoplasmic extracts, respectively. (**C**) Western blot analysis showed higher expression levels of phosphoserine FOXG1 (arrow) in SKOV3 and A2780cp cell lines. (**D**) An increased phosphoserine FOXG1 was detected in Flag-tagged FOXG1 stable clone (Acp-C4) as compared with vector control (Acp-C4) of A2780cp.

It has been shown that FOXG1 is able to block p21^WAF1/CIP1^ induction by interacting with FoxO–Smad complexes in nucleus (Seoane *et al*, 2004). To examine whether the inhibition of p21^WAF1/CIP1^ induction in overexpressed FOXG1 ovarian cancer cells was due to the increased accumulation of nuclear FOXG1, we conducted western blot analysis on subcellular extracts from ovarian cancer cell lines. Of four ovarian cancer cell lines, a relatively higher level of nuclear FOXG1 was observed in SKOV3 and A2780cp cells, which expressed relatively higher levels of FOXG1 as compared with OVCA420 and OVCA429 cells (Figures 1A and 7A). We also found that a significant increase of nuclear Flag-tagged FOXG1 was observed in Flag-tagged FOXG1 stably expressing clone (Acp-C4) (Figure 7B). Conversely, a reduction in nuclear FOXG1 was found in FOXG1 knockdown clone (Acp-shC6) of A2780cp cells (Figure 7B). A recent finding has shown that the elevation of phosphorylation at Ser19 of FOXG1 promotes nuclear import (Regad *et al*, 2007). Thus, we attempted to evaluate the serine phsophorylation levels of FOXG1 according to the similar protocol of Regad *et al* (2007). By immunopreciptation and western blot assays, an increase level of serine phosphorylation of FOXG1 was detected in SKOV3 and A2780cp cells, and in Flag-tagged FOXG1 stably expressing clone (Acp-C4) (Figure 7C and D). These results show that the inhibition of p21^WAF1/CIP1^ induction in FOXG1-overexpressed ovarian cancer cells is due to the increased serine phosphorylation and nuclear localisation of FOXG1.

## Discussion

Loss of responsiveness to the growth inhibitory effect of TGF-*β* is a substantial mechanism in cancer development (Moses *et al*, 1987). Apart from genetic and/or epigenetic lesions in TGF-*β* receptors or TGF-*β*/Smad transducers that can contribute to TGF-*β* resistance in human cancers (Arai *et al*, 1998; Ko *et al*, 1998; Matsushita *et al*, 1999; Venkatasubbarao *et al*, 2000; Maurice *et al*, 2001; Fukushima *et al*, 2003; Sakaguchi *et al*, 2005; Zhong *et al*, 2006), emerging data have also suggested that the expressions of viral proto-oncogenes and oncogenes are capable of inducing TGF-*β* resistance through blocking the functions of Smads (Datta and Bagchi, 1994; Hahm *et al*, 1999; Mori *et al*, 2001; Lee *et al*, 2002; Dowdy *et al*, 2003; He *et al*, 2003; Wang *et al*, 2008). In this study, we found that FOXG1 was overexpressed in ovarian cancer cell lines and tissue samples. Using ovarian cancer cell models, we showed that overexpressed FOXG1 could suppress the TGF-*β*/Smad pathway-induced p21^WAF1/CIP1^ expression and enhanced cell proliferation in ovarian cancer cells. These data suggest that FOXG1 is another oncogene participating in TGF-*β* resistance through suppressing p21^WAF1/CIP1^ expression mediated by TGF/Smad signalling in ovarian cancer cells.

FOXG1 is highly expressed in the telencephalon and has a crucial role in both proliferation and differentiation of neocortical progenitors during brain development (Hanashima *et al*, 2002; Muzio and Mallamaci, 2005). Indeed, *v-Qin* and *c-Qin*, the orthologs of FOXG1, have been reported to induce oncogenic transformation of chicken embryo fibroblasts, suggesting FOXG1 may function as an oncogene in human cancer (Chang *et al*, 1996; Li *et al*, 1997). This is further evidenced by the current reports that overexpression of FOXG1 is associated with the development of human medulloblastoma and glioblastoma through abolishing TGF-*β*-mediated growth inhibition by suppressing the transcription activation of *p21*^*WAF1/CIP1*^ (Seoane *et al*, 2004; Adesina *et al*, 2007b). However, these reports just indicate the important roles of FOXG1 in the development of brain cells and neural tumourigenesis. The roles and the oncogenic potential of FOXG1 are rarely reported in other human epithelial-derived cancers. In this study, we showed that FOXG1 exercises an oncogenic function in attenuating the anti-proliferative control of TGF-*β* through negative regulation of p21^WAF1/CIP1^ expression in ovarian cancer cells. This suggests that the oncogenic role of FOXG1 contributing to TGF-*β* resistance is not only restricted in human medulloblastoma and glioblastoma but also extends to other human epithelial-derived cancers.

TGF-*β* exerts cell growth inhibitory effects through induction of the CDK inhibitors p15^Ink4b^ and p21^WAF1/CIP1^ expressions through Smad-mediated transcriptional activities (Voss *et al*, 1999; Feng *et al*, 2000). These inhibitors are able to block cyclin and CDKs from phosphorylating the retinoblastoma protein (Rb), as well as preventing the progression of the cell cycle (Reynisdottir *et al*, 1995; Massague *et al*, 2000). FOXG1 has been shown to inhibit Smad-mediated induction of p21^WAF1/CIP1^ expression through association with FoxO–Smad complexes in the nucleus (Rodriguez *et al*, 2001; Seoane *et al*, 2004; Adesina *et al*, 2007b). To investigate the cell growth inhibitory mechanism of FOXG1 on ovarian cancer cells, we evaluated the p21^WAF1/CIP1^ expression by quantitative RT–PCR, western blot and immunohistochemical analyses. Although we observed that there was an inverse relationship between the expressions of FOXG1 and p21^WAF1/CIP1^, no significant statistical correlation was found. However, this is not surprising because p21^WAF1/CIP1^ is usually downregulated in human cancers and is regulated by p53-dependent and -independent pathways (O'Reilly, 2005). Therefore, to exclude the effect of p53 on p21^WAF1/CIP1^ expression, we used *p21*^*WAF1/CIP1*^ promoter (truncated without p53-binding site) luciferase reporter assay to study the effect of FOXG1 on *p21*^*WAF1/CIP1*^ in HEK293T and A2780cp cell models. Consistent with the findings from FOXG1 on human medulloblastoma and glioblastoma (Rodriguez *et al*, 2001; Seoane *et al*, 2004; Adesina *et al*, 2007b), FOXG1 could reduce TGF-*β*-mediated p21^WAF1/CIP1^ expression in a dose-dependent manner. We also showed that the overexpression or RNAi-mediated depletion of FOXG1 could alter the expression of p21^WAF1/CIP1^ in ovarian cancer cells. This suggests that FOXG1 is able to inhibit TGF-*β*-mediated p21^WAF1/CIP1^ induction and supports its growth promoting function in ovarian cancer cells.

Recent studies have shown that the phosphorylation of Ser 19 at the N-terminus of FOXG1 promotes nuclear imports of FOXG1 (Regad *et al*, 2007). The nuclear FOXG1, in turn, blocks TGF-*β*-mediated p21^WAF1/CIP1^ induction (Seoane *et al*, 2004). To investigate whether the inhibitory effect on p21^WAF1/CIP1^ induction is due to this mechanism, we evaluated subcellular localisation and serine phosphorylation status of FOXG1. Our data showed that higher levels of serine phosphorylation of FOXG1 were consistent with the accumulation of nuclear FOXG1 in FOXG1-overexpressed ovarian cancer cell lines (SKOV3 and A2780cp) and Flag-tagged FOXG1 enforced-expressing cells. However, no change of FOXG1 nuclear localisation and serine phosphorylation levels were observed in ovarian cancer cells under TGF-*β* treatment (see Supplementary Figure). This indicates that the increased serine phosphorylation and nuclear localisation of FOXG1 in FOXG1-overexpressed ovarian cancer cells attribute the inhibition of TGF-*β*-mediated p21^WAF1/CIP1^ induction.

Finally, apart from inhibition on cell proliferation, TGF-*β* also induces differentiation and apoptosis in many normal epithelial cells (Elliott and Blobe, 2005). Loss of TGF-*β* responsiveness has been found in high grade with poor differentiated human cancers (Tang *et al*, 2003; Elliott and Blobe, 2005). Intriguingly, the overexpression of FOXG1 was also significantly correlated with a higher grade of ovarian cancer and in agreement with the above findings. These findings suggest that overexpressed FOXG1 suppresses TGF-*β* responsiveness in ovarian cancer.

## Figures and Tables

**Table 1:** Clinicopathological correlation of FOXG1 expression in ovarian cancer patients

		**FOXG1 expression (fold)**		
**Characteristics**	**Total**	**⩽1.5**	**>1.5**	***P***
All cases	67	33 (49.25%)	34 (50.75%)	
				
*Age (y)*				
<55	39	16 (41.03%)	23 (58.97%)	
>55	28	17 (60.71%)	11 (39.29%)	0.112
				
*Stage*				
Early	21	12 (57.14%)	9 (42.86%)	
Late	43	21 (48.84%)	22 (51.16%)	0.532
				
*Grade*				
1 and 2	20	14 (70.00%)	6 (30.00%)	
3	29	11 (37.93%)	18 (62.07%)	0.027^*^
				
*Histological subtypes*				
Serous+/−papillary	21	13 (61.90%)	8 (38.10%)	
Others	46	20 (43.48%)	26 (56.52%)	0.162
Mucinous	14	5 (35.71%)	9 (64.29%)	
Others	53	28 (52.83%)	25 (47.17%)	0.255
Endometrioid	17	7 (41.18%)	10 (58.82%)	
Others	50	26 (52.00%)	24 (48.00%)	0.441
Clear cells/undifferentiated	15	8 (53.33%)	7 (46.67%)	
Others	52	25 (48.08%)	27 (51.92%)	0.720
				
*Recurrence*				
+	31	15 (48.39%)	16 (51.61%)	
−	31	16 (51.61%)	15 (48.39%)	0.799

**Table 2:** Immunohistochemical scores of FOXG1 on tissue array of ovarian cancer

		**Expression level (%)**		
**Histological types**	**Number of cases**	**Negative**	**1+/2+**	**3+/4+**
Normal/benign tumour	6	5 (58.3)	1 (16.7)	0
Clear cell/undifferentiated	3	2 (66.7)	0	1 (33.4)
Serous	9	0	2 (22.2)	7 (77.8)
Mucinous	6	2 (33.3)	3 (50.0)	1 (16.7)
Endometrioid	13	2 (15.4)	2 (15.4)	9 (69.4)
