# Supplementary figures and images for: Overexpression of FOXG1 contributes to TGF-β resistance through inhibition of p21WAF1/CIP1 expression in ovarian cancer
Source: Br J Cancer. 2009 Sep 15;101(8):1433–43. doi: 10.1038/sj.bjc.6605316 (PMC2768441; doi:10.1038/sj.bjc.6605316)

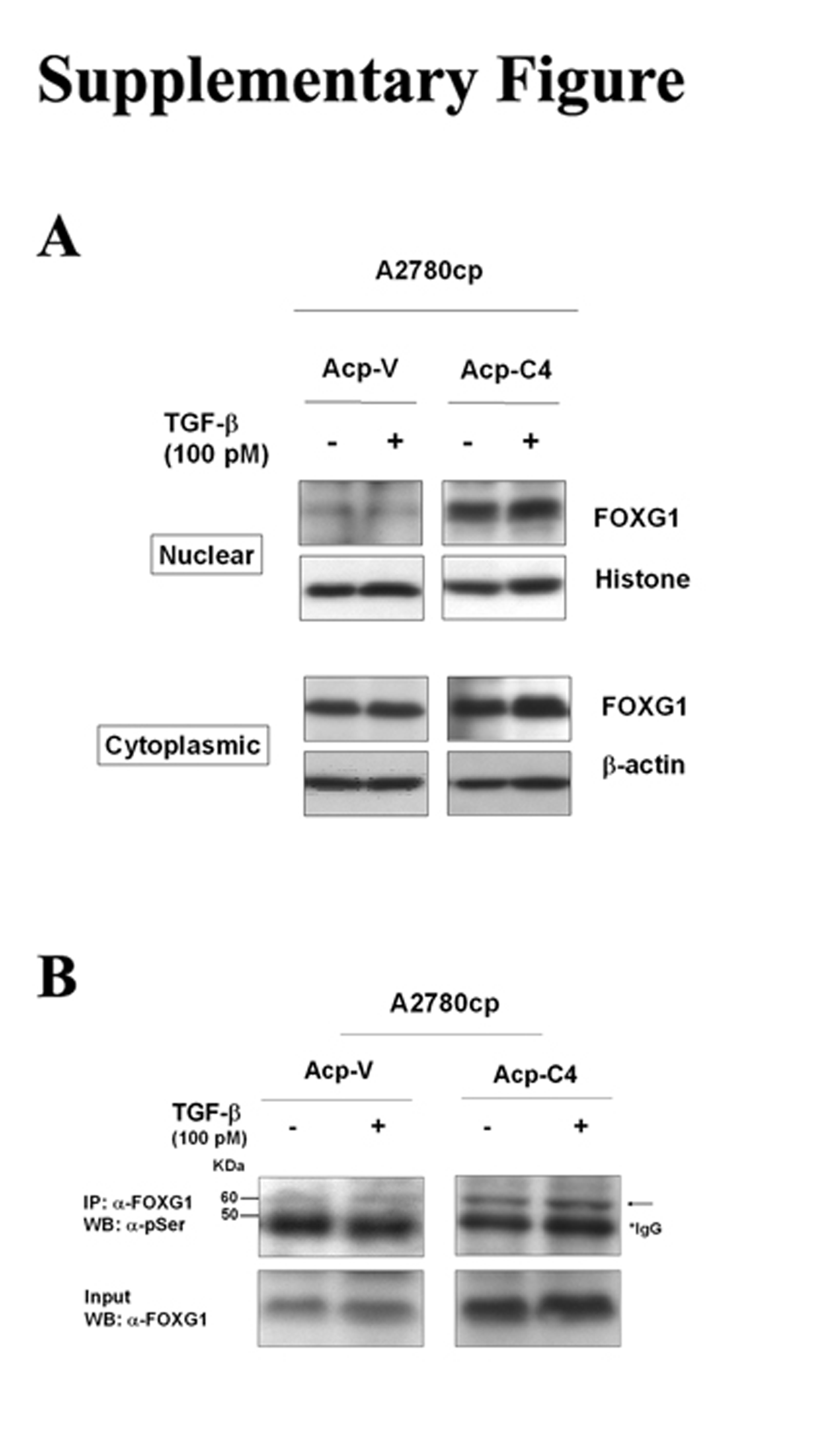

Supplement: Supplementary Figure 1 [file 6605316x1.tif]
