# Supplementary material for: Overexpression of FOXG1 contributes to TGF-β resistance through inhibition of p21WAF1/CIP1 expression in ovarian cancer
Source: Br J Cancer. 2009 Sep 15;101(8):1433–43. doi: 10.1038/sj.bjc.6605316 (PMC2768441; doi:10.1038/sj.bjc.6605316)
Supplement: Supplementary Figure Legend [file 6605316x2.doc]

**Supplementary Figure Legend.** TGF-β treatment did not alter subcellular localization and phosphorylation of FOXG1. (A) Western blot analysis showed that there was no difference of cytoplasmic and nuclear FOXG1 expressions between vector control (Acp-V) and Flag-tagged FOXG1 stable clone (Acp-C4) of A2780cp cells. Histone H1 and β-actin were used as internal controls of nuclear and cytoplasmic extracts respectively. (B) Western blot analysis showed no difference of phosphoserine FOXG1 levels (*arrow*) between vector control (Acp-V) and Flag-tagged FOXG1 stable clone (Acp-C4) of A2780cp cells.
